# Phylogeny and Pathogenicity of Subtype XIIb NDVs from Francolins in Southwestern China and Effective Protection by an Inactivated Vaccine

**DOI:** 10.1155/2023/1317784

**Published:** 2023-04-05

**Authors:** Tingting Zeng, Liji Xie, Zhixun Xie, Jiaoling Huang, Zhiqin Xie, Qinghong Huang, Sisi Luo, Sheng Wang, Meng Li, Jun Hua, Yanfang Zhang, Minxiu Zhang

**Affiliations:** ^1^Guangxi Key Laboratory of Veterinary Biotechnology, Guangxi Veterinary Research Institute, Nanning 530000, Guangxi, China; ^2^Key Laboratory of China (Guangxi)-ASEAN Cross-Border Animal Disease Prevention and Control, Ministry of Agriculture and Rural Affairs of China, Guangxi Veterinary Research Institute, Nanning 530000, Guangxi, China; ^3^College of Animal Science and Technology, Guangxi University, Nanning 530000, Guangxi, China

## Abstract

Most genotype XII newcastle disease viruses (NDVs) were isolated from poultry, chickens, or geese, with the exception of one subtype, XIIa NDV, which was isolated from a peacock. Here, two subtype XIIb NDVs, francolin/China/GX01/2017 and francolin/China/GX02/2017 (GX01 and GX02 hereafter), were isolated from francolins, which are resident birds in southern China. GX01 and GX02 were characterized as velogenic NDVs. Based on the weaker pathogenicity of these viruses in chickens, the amino acid sequences of seven proteins from genotype XII NDVs were compared, which revealed 17, 40, 15, 7, 32, 25, and 31 variations in the NP, P, M, F, HN, L, and V proteins, respectively, some of which could be responsible for this decreased pathogenicity. Epidemiological and phylogenetic analyses suggest that subtype XIIb NDVs have multiple transmission chains, and that resident birds may be involved in this process as intermediate hosts in which viruses keep evolving. Because of the increased pathogenicity of subtype XIIb NDVs, the protective efficacy of GX01 as an inactivated vaccine was evaluated and compared with that of two commercial inactivated vaccines in chickens. The results showed that the subtype XIIb NDVs could be candidate genotype-matched vaccine strains against genotype XII NDVs.

## 1. Introduction

Newcastle disease viruses (NDVs), often referred to as avian paramyxoviruses 1 (APMV-1), which have been classified to the genus Avian orthoavulavirus 1 (AOAV-1) within the family Paramyxoviridae and cause diseases in a variety of domestic and wild birds worldwide [1]. Virulent strains, which are highly contagious and pathogenic, are viruses that have an intracerebral pathogenicity index (ICPI) of at least 0.7 (2.0 is the maximum) or a fusion cleavage site with several basic amino acids and phenylalanine at position 117 [2]. Currently, NDVs are divided into two classes, class I with subtypes 1.1.1, 1.1.2, and 1.2, and class II with at least 20 genotypes (I to XXI) in class II according to new naming criteria [3]. Epidemiological data have revealed that genotypes VI and VII NDVs are the predominant epidemic strains in China and cause substantial poultry industry financial losses [4–7]. Genotype XII NDVs were isolated and characterized in 2010 in Guangdong Province, China [8].

Genotype XII NDVs isolated in China cluster in subtype XIIb [8], whereas subtype XIIa NDVs were isolated in South America, and subtype XIId NDVs were isolated in Vietnam [9–11]. Subtype XIIa and XIId NDVs have all been isolated from birds with typical ND manifestations, and the fusion cleavage site or the ICPI value indicated that these strains belonged to velogenic NDVs and caused lethality in chickens in experiments [10]. However, no outbreak associated with this genotype has been reported in China, and subtype XIIb NDVs have been isolated from apparently healthy geese since 2010. In this study, two subtype XIIb NDV strains were isolated from apparently healthy francolins. Although the fusion cleavage site or the ICPI value indicated that all subtype XIIb NDVs were velogenic NDVs, subtype XIIb NDVs caused ND manifestations but not death in chickens in both previous studies [8] and our work. Due to the differences in pathogenicity, the variations in amino acids of seven proteins (NP, P, M, F, HN, L, and V) were examined. The protection efficiency of inactivated vaccines of genotype XII NDVs with commercial vaccines were evaluated.

## 2. Materials and Methods

### 2.1. Virus Isolation and Pathogenic Evaluation

The NDVs francolin/China/GX01/2017 and francolin/China/GX02/2017 (abbreviated GX01 and GX02, respectively, below) were isolated from oropharyngeal and cloacal swabs of apparently healthy francolins in the suburban region of Nanning city, Guangxi Province, southwestern China, during a surveillance project in 2017. Oropharyngeal and cloacal suspend oropharyngeal and cloacal swab samples in 1 mL of phosphate-buffered saline (PBS) containing antibiotics (penicillin, 1000 U/mL; streptomycin, 100 *μ*g/mL; amphotericin B, 0.25 *μ*g/mL). Virus isolation was carried out using 9-day-old SPF chicken embryos (Beijing Merial Vital Laboratory Animal Technology Co., Ltd., China) and then incubated at 37°C.

Allantois fluids were harvested and identified by the hemagglutination (HA)-inhibition (HI) test following the OIE protocol [2]. Briefly, the HA titers of allantois fluids were determined with 0.5% chicken red blood cells (RBCs) then were generated against 4 hemagglutination units of standardized antigen (4U antigen). Antisera of NDV, avian influenza virus (AIV) subtypes H1–H16, and egg drop syndrome virus (EDSV) were serially double diluted and mixed with 4U of antigen. RBCs were added to assess the HI titers. Viruses were identified when HA was blocked by the corresponding antiserum in the HI assay.

After four plaque purifications in DF-1 cells, the viruses were propagated in 9-day-old SPF chicken embryos and stored at −80°C until use. The virulence of the NDVs was evaluated by determining the mean death time (MDT) in 10-day-old SPF chicken embryos and intracerebral pathogenicity test (ICPI) in 1-day-old SPF chicks following the OIE protocol [2].

### 2.2. Genome Sequencing

Viral genomic RNA was extracted with a viral RNA/DNA extraction kit. Complete genomes were amplified by one-step RT-PCR with the High Fidelity One Step RT-PCR Kit and previously described primers, [12] according to the kit's protocol. RT-PCR fragments were amplified, ligated into a pMD18-T vector then transformed into *Escherichia coli* DH5*α*. All reagents and kits were purchased from Takara (Takara, Dalian, China). The positive clones were sequenced by BGI (BGI, Shenzhen, China). The two genomes were assembled by using the SeqMan program (DNASTAR, Inc. Madison, USA). The GenBank accession numbers of GX01 and GX02 are MZ306226 and MZ306225, respectively.

### 2.3. Genome Analysis

The deduced amino acid sequences of all protein-encoding genes were compared with those of the other genotype XII NDVs by the MegAlign program (DNASTAR, Inc.). To analyze the evolutionary relationship, a maximum likelihood tree was constructed with a pilot tree dataset using full-length F gene sequences [3]. The maximum likelihood tree was constructed via the CIPRES Science Gateway website [13] based on a general time-reversible (GTR) model with the combination GTR + Γ + I and 1000 bootstrap replicates. The mean evolutionary distances between groups were calculated by MEGA v. X software based on the number of base substitutions per site from averaging over all sequence pairs between groups utilizing the maximum composite likelihood model with rate variation among sites with a gamma distribution (shape parameter = 1). Trees were visualized using FigTree v1.4.2 (https://tree.bio.ed.ac.uk/software/figtree).

### 2.4. Estimated Protection Efficiency of an Inactivated Vaccine of Genotype XII NDVs Compared with Commercial Vaccines

#### 2.4.1. Inactive Vaccine Generation

The protective efficacy of the commercial vaccines in China, LaSota and A-VII [14], were evaluated in a previous study [8]. In this study, GX01 was used as an inactivated vaccine for comparison with the protective efficacy of LaSota and A-VII. GX01 was propagated in 9-day-old SPF chicken embryos, and allantois fluids were harvested. The allantois fluids were filtered and concentrated by sucrose gradient centrifugation to a 10 log_2_ HA titer. Viral suspensions were inactivated by incubation with 0.02% *β*-propiolactone (Sigma-Aldrich, Darmstadt, Germany) at 4°C for 24 h and then mixed with Tween 80 at a 9 : 1 ratio to constitute the aqueous phase. Lipids mixed with Span 80 at a 25 : 1 ratio constituted the oil phase. The aqueous phase was mixed with the oil phase at a 1 : 4 ratio to generate the inactive vaccine. PBS was used to replace GX01 to generate an oil-emulsion vaccine as a control. All reagents used to generate the oil-emulsion vaccine were purchased from Sigma (Sigma-Aldrich, Darmstadt, Germany).

#### 2.4.2. Animal Experiments

SPF chickens were divided into four groups and immunized with the inactivated vaccines GX01, LaSota, and A-VII or PBS. Each chicken was immunized twice, at two weeks old and four weeks old, with a volume of 0.2 ml. The antibody titers were monitored every week using the corresponding antigen by HI assay as descripted in Section 2.1. Five weeks after the first immunization, eleven SPF chickens with antibody titers reaching 10 log_2_ were selected for challenge with 10^6^ EID_50_ in a volume 0.2 ml of GX01 through intraocular and intranasal routes, and six SPF chickens in the PBS-immunized group were challenged with 0.2 ml PBS through the same routes.

#### 2.4.3. Detection of Viral Shedding

All birds were observed daily for clinical symptoms for 14 days. To detect viral shedding, oropharyngeal and cloacal swabs were collected at 1, 3, 5, 7, 9, 11, and 13 days post infection (dpi). The swab samples were suspended in 1 ml of PBS containing antibiotics as described before, and then viral titers of swab samples were determined by a 50% egg infectious dose (EID_50_) assay and calculated by the Reed and Muench's method.

#### 2.4.4. Pathological Observation and Analysis

In addition, three SPF chickens in each group were randomly selected and dissected at 3 dpi for observation, and tissue lesions of the liver, spleen, kidney, lung, bursa, pancreas, glandular stomach, cecum tonsil, and brain were collected and fixed in a 10% formalin solution to prepare pathological sections. The collected tissues and organs were fixed in a 10% neutral formalin solution for more than 24 h, dehydrated and cleared with an alcohol series. After paraffin sealing, the tissues were embedded, and 4–10 *μ*m-thick sections were prepared. After HE staining, the sections were dehydrated and sealed, and pathological changes in the sections were observed by microscopy (Nikon, Tokyo, Japan) and image capture.

### 2.5. Statistical Analysis

Data were analyzed by using Prism (v.9.4.0) using the ROUT test (GraphPad Software Inc., USA). The D'Agostino-Pearson normality test was used to estimate whether the values in each group were derived from according with a Gaussian distribution. One-way ANOVA statistical analysis was used for multiple comparisons of viral titers in oropharyngeal and cloacal swab samples from each group with the same sampling point based on the normality distribution. The statistical significance threshold was set at a *P* value of <0.05.

## 3. Results

### 3.1. Isolation and Characterization of NDVs

The NDVs GX01 and GX02 were HI positive with NDV-specific antiserum but negative with antisera for avian influenza viruses (AIVs) of subtypes H1–H16 and egg drop syndrome virus (EDSV) [15]. The MDT values of GX01 and GX02 were 58 h and 57 h, and the ICPI values were 1.613 and 1.65, respectively. According to the OIE protocol, GX01 and GX02 were characterized as velogenic NDVs.

### 3.2. Genome Sequencing and Phylogenetic Analyses with a Pilot Tree

The lengths of both GX01 and GX02 were 15192 nt, composing six genes in the order 3′-*NP*-*P*-*M*-*F*-*HN*-*L*-5′. Both strains had leader and trailer sequences comprising 55 and 114 nt, respectively. The length of the intergenic region of the *NP*-*P*, *P*-*M,* and *M*-*F* genes was 1 nt and that of the *F*–*HN* and *HN*-*L* genes was 31 and 47 nt, respectively.

**Figure 1:**
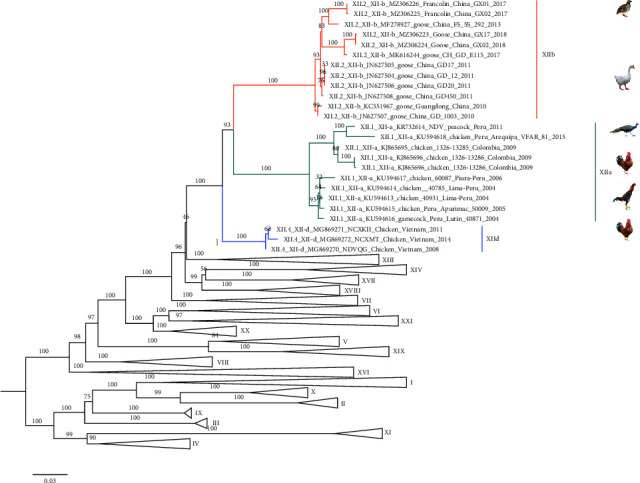
Maximum likelihood tree of the full-length *F* gene with the class II pilot tree plus all genotype XII NDVs reported previously. The maximum likelihood tree was constructed via the CIPRES science gateway based on a general time-reversible (GTR) model with the combination GTR + Γ + I and 1000 bootstrap replicates. The strains of subtype XIIa are shown in green, subtype XIId is shown in blue, GX01 and GX02 are shown in purple, and the other genotype II subtype XIIb NDVs are shown in orange. The other NDVs of class II were collapsed for better illustration.

A maximum-likelihood tree was constructed based on the full length of the *F* gene with the pilot tree plus all genotype XII NDVs as reported previously (Figure 1). The distance between GX01 and GX02 was 0.0006. These two strains were clustered with subtype XIIb NDVs isolated in China, and the distance from other subtype XIIb NDVs was 0.0174–0.0220. The average genetic distance between Chinese strains (subtype XIIb) and South American (subtype XIIa) strains was 0.0909, and it was 0.0691 between Chinese strains (subtype XIIb) and Vietnam strains (subtype XIId).

### 3.3. Analysis of Six Protein-Encoding Genes

The open reading frame (ORF) of the *F* gene of these two strains was 1662 nt in length, encoding 553 amino acids (aa). The deduced amino acid at the cleavage site of the protein *F* was 112RRQKR↓F117, which is the determinant of velogenic NDVs. There were 7 deduced amino acid variations between subtype XIIb and XIIa, XIId NDVs, with 1 variation in the signal peptide, 3 in heptad repeat regions, 1 in the cytoplasmic tail, and 2 in other regions [16] (Supplement Table 1). Six N-linked glycosylation sites [17] were conserved among the strains and were analyzed.

The ORFs of the *HN* gene of these two strains were 1716 nt in length, encoding 571 amino acids (aa). HN protein sequences extracted from four subtype XIIb NDVs from Guangxi Province, two from Guangdong Province, China, and two subtype XIIa NDVs were analyzed since only *F* gene information is available in GenBank for the remaining genotype XII NDVs. Comparing the HN protein sequences of genotype XII NDVs showed 5, 5, 2, and 20 variations in the cytoplasmic tail [18], transmembrane domain [19], stalk region, and globular head [20, 21], respectively (Supplement Table 2, only residues conserved among subtype XIIb and different from subtype XIIa NDVs are shown and are the same with the following protein). Ten residues of the first sialic acid binding site [22] and eight conserved residues of the second sialic acid binding site [23] were conserved among genotype XII NDVs. Six N-linked glycosylation sites (119, 341, 433, 481, 508, and 538) were predicted in most NDVs; all subtype XIIb NDVs lost site 538, NDV/peacock/Peru/2011 lost sites 508 and 538, and chicken/Peru/1918-03/603/2008 lost site 508.

Regarding the remaining proteins, the *P* gene encoded 395 amino acids (aa) and encoded a V protein with 239 amino acids after insertion of one G residue at the conserved editing site (UUUUUCCC, genome sense). There were 40 and 31 amino acid variations in the proteins P and V between subtype XIIb and XIIa NDVs, respectively. The *NP*, *M*, and *L* genes encoded 489, 364, and 2204 amino acids (aa) and shared 17, 15, and 25 amino acid variations between subtype XIIb and XIIa NDVs, respectively (Supplement Tables 3–7).

Upon comparing seven neutralizing epitopes of protein F located at residues 72, 74, 75, 78, 79, 157 to 171, and 343 [24] with those of the vaccine strains LaSota and A-VII [25], subtype XIIb NDVs had a substitution of D to N at residue 170 as well as in the HR1 region, whereas the remaining neutralizing epitope residues were conserved with LaSota and A-VII. As there were seven overlapping antigenic sites of protein HN (sites 1: 345; site 2: 513, 514, 521, and 569; site 3: 236, 287, and 321; site 4: 332, 333, and 356; site 12: 494, and 516; site 14: 347, 350, and 353; site 23: 193, 194, and 201) [26, 27] with those of vaccine strains, two residues (347 D and 521 N) differed from those in A-VII and LaSota (347 E and 521 S) (Supplement Table 8).

In addition, 17 variations were found between francolin strains and other genotype XII NDVs: 4 variations in protein F, 3 in protein HN, 1 in protein NP, 1 in protein L, 4 in protein *P,* and 4 in protein V (Supplement Table 9). Among these variations, 2 in transmembrane domain and 1 in globular head of protein HN. Four variations in the amino acid compositions of the proteins were found between GX01 and GX02 genomes; these amino acids are residue 474 (I in GX01 and M in GX02) of protein F, residue 171 (G in GX01 and S in GX02) of protein HN, and residues 717 (V in GX01 and I in GX02), and 1603 (I in GX01 and T in GX02) of protein *L*.

### 3.4. Comparison of the Inactivated Genotype XII NDV Vaccine with Commercial Vaccines

**Figure 2:**
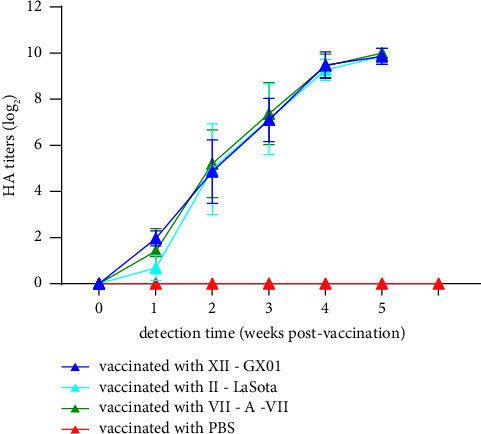
Antibody titers in the four groups inoculated with different vaccines. The data are presented as the means and SDs. Error bars indicate the standard deviations of measurements of 29 blood samples (*n* = 29).

**Figure 3:**
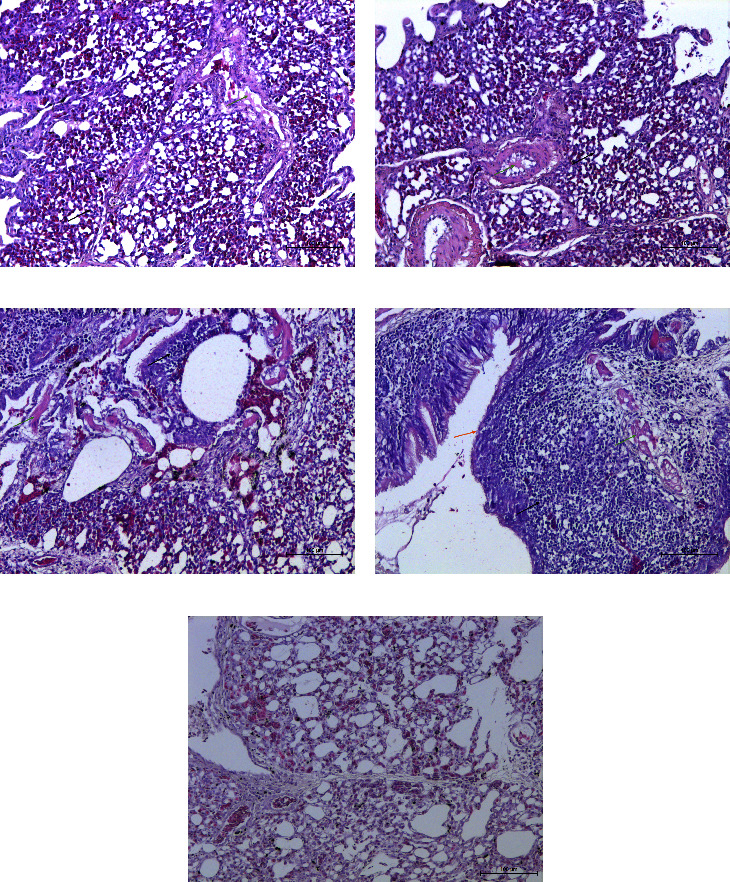
Pathological observation of the lungs. Black arrows indicate infiltrated inflammatory cells, green arrows indicate inflammatory exudate, and orange arrows indicate detached epithelial cells.

**Figure 4:**
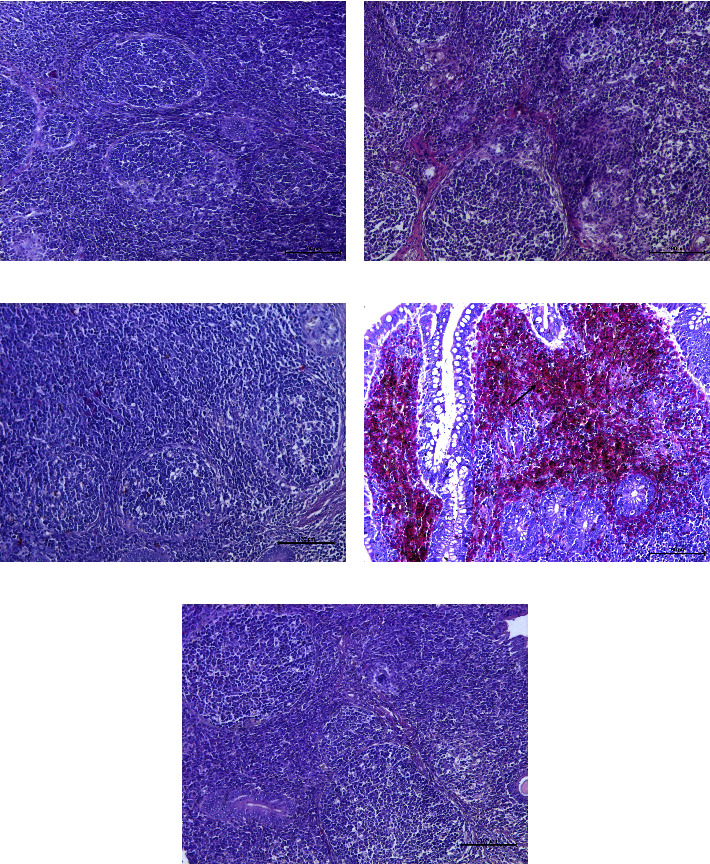
Pathological observation of the cecal tonsil. Black arrows indicate hemorrhage.

The antibody titers of each chicken was monitored every week after immunization and reached 9 log_2_-10 log_2_ at four weeks after the first immunization, whereas the titers of the PBS-immunized group remained negative (Figure 2). Challenge with GX01 was carried out five weeks after the first immunization. Chickens in the GX01-, LaSota-, and A-VII-immunized groups did not show any obvious clinical symptoms throughout the 14-day observation time frame, and chickens in the PBS-immunized group showed slight depression, ruffled feathers, diarrhea, and rhinorrhea between 5 and 10 days post infection (dpi). Examination of histopathologic slides from 9 organs at 3 dpi showed that the bronchiolar mucosa was edematous, with a large number of infiltrated inflammatory cells and some detached epithelial cells, and the lumen of the lung was filled with inflammatory exudate in the PBS-immunized group (Figure 3(d)). A small number of inflammatory cells had infiltrated the bronchioles, and inflammatory exudate was observed in the GX01/LaSota/A-VII-immunized group (Figures 3(a)–3(c)). The normal lung of a chicken inoculated with PBS is shown in Figure 3(e). Cecal tonsil hemorrhage was observed in the PBS-immunized group (Figure 4(d)) but not the GX01/LaSota/A-VII-immunized group (Figures 4(a)–4(c)). A normal cecal tonsil from a chicken inoculated with PBS is shown in Figure 3(e). The rest of the organs in each group showed no apparent lesions.

**Figure 5:**
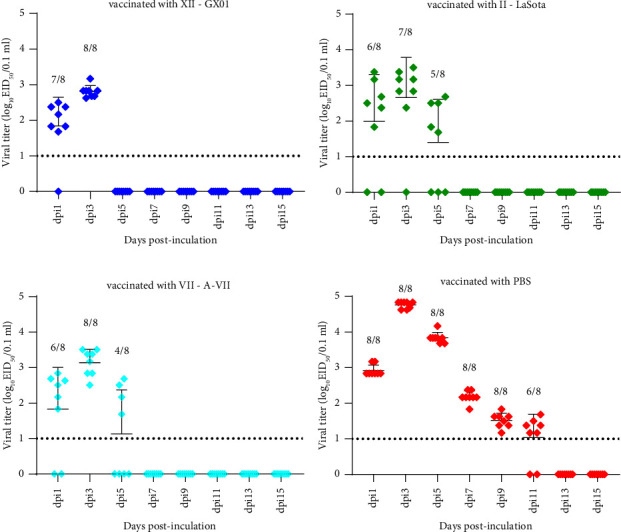
Viral titers cloacal swabs after inoculating with GX01. The data are presented as the individual values, means, and SDs. Above each time point, the ratio of swabs was displaying positive recovery to the number of all tested swabs in each group at the sampling time points. The error bars indicate the standard deviations of measurements for 8 swabs (*n* = 8). The dashed lines represent the lower limits of detection.

**Figure 6:**
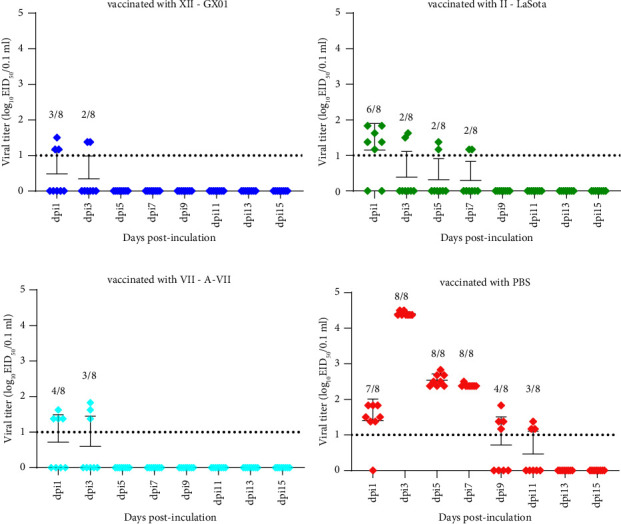
Viral titers in oropharyngeal swabs after inoculating with GX01. The data are presented as the individual values, means, and SDs. Above each time point, the ratio of swabs was displaying positive recovery to the number of all tested swabs in each group at the sampling time points. The error bars indicate the standard deviations of measurements for 8 swabs (*n* = 8). The dashed lines represent the lower limits of detection.

Oropharyngeal and cloacal swabs were collected for the EID_50_ assay to evaluate viral shedding at 1, 3, 5, 7, 9, 11, 13, and 15 dpi. In the GX01-immunized group, viral shedding was detected at 1 and 3 dpi (7/8 and 8/8) via the oropharyngeal route, with titers ranging from 1.681 to 2.833 log_10_EID_50_/0.1 ml, and via the cloacal route (3/8 and 2/8), with titers ranging from 1.168 to 1.5 log_10_EID_50_/0.1 ml (Figures 5(a) and 6(a)). In the LaSota-immunized group, virus titers varied from 1.681 to 3.5 log_10_EID_50_/0.1 ml at 1, 3, and 5 dpi (6/8, 7/8, and 5/8) in the oropharyngeal route and 1.167 to 1.833 log_10_EID_50_/0.1 ml at 1, 3, 5, and 7 dpi (6/8, 1/8, 2/8, and 2/8) in the cloacal route (Figures 5(b) and 6(b)). In the A-VII-immunized group, viral shedding was detected at 1, 3, and 5 dpi (6/8, 8/8, and 4/8) via the oropharyngeal route, with titers ranging from 1.681 to 3.5 log_10_EID_50_, and at 1 and 3 dpi via the cloacal (4/8 and 3/8) route, with titers ranging from 1.375 to 1.833 log_10_EID_50_ (Figures 5(c) and 6(c)). In the PBS-immunized group, viral shedding was detected from 1 to 11 dpi, in the oropharyngeal route (8/8 from 1 to 9 dpi and 6/8 at 11 dpi), titers varied from 1.167 to 4.833 log_10_EID_50_, and in the cloacal route (7/8, 8/8, 8/8, 8/8, 4/8, and 3/8) with 1.167 to 4.5 log_10_EID_50_ (Figures 5(d) and 6(d)). The EID_50_ values from the oropharyngeal and cloacal swabs are listed in Supplement Tables 10 and 11.

Viral shedding titers in both routes in the GX01-immunized group were significantly lower than PBS-immunized group but not significantly different between the LaSota-immunized and A-VII-immunized groups at 1 dpi. The viral shedding titers of the three vaccine groups in both routes were significantly lower than PBS-immunized group at 3, 5, and 7 dpi, and there were no significant differences among them (Table 1).

## 4. Discussion

Subtype XIIa NDVs were isolated and characterized in 2004 in Peru [10], and they caused severe clinical manifestations and up to 100% mortality in gamecocks. Then, infection spread to vaccinated layers (with a 1.8% mortality rate, minor respiratory difficulties, and decreased egg production) and broilers (with 4%–12% mortality, respiratory difficulties, and depression). A strain from a peacock was isolated in 2011 and caused 90% mortality of a peacock flock, and killed 100% of nonvaccinated SPF chickens after inoculation with 2 × 10^2^ PFU [9]. On the other hand, subtype XIId NDVs have been isolated and characterized in Vietnam since 2008 and have caused typical clinical manifestations and death in infected chickens. Subtype XIIb NDVs of China have been isolated in apparently healthy geese since 2010 [8, 28]. The MDT, ICPI and cleavage site of protein *F* indicated that all genotype XII NDVs were velogenic, but subtype XIIb caused only clinical manifestations with 0% mortality after inoculation with 10^6^ EID50 of strain E115 via intraocular and intranasal routes in 6-week-old SPF chickens, and artificially infected geese exhibited no clinical manifestations [8]. In this study, GX01 and GX02 were isolated from apparently healthy francolins, and two other strains, goose/China/GX02/2018 and goose/China/GX17/2018, were isolated from apparently healthy geese. The MDT, ICPI, and cleavage site of protein F of these four strains indicated that they are velogenic NDVs. Seven-week-old SPF chickens were challenged with 10^6^ EID_50_ of GX01 through intraocular and intranasal routes, which caused slight depression, ruffled feathers, diarrhea, and rhinorrhea without death. It is interesting that these velogenic NDVs from China were not lethal to SPF chickens and showed significant pathogenicity differences from subtype XIIa and XIId NDVs. Examination of histopathologic slides from 9 organs at 3 dpi showed that there was an inflammatory response in the lungs, hemorrhage in the cecal tonsil in PBS-immunized group, yet the rest of the organs in each group showed no apparent lesions. It is demonstrated that the lesion were milder than other velogenic NDVs [29].

All seven proteins and noncoding regions contributed to the virulence of NDV, whereas proteins F, HN, and V majorly contribute to virulence according to a compilation of the data [29]. Although the deduced amino acids of the cleavage site in protein F were characterized as a major molecular determinant of NDV virulence [30, 31], some other functional regions in protein F may also influence NDV pathogenicity, as fusogenic ability is responsible for virulence. For example, nonconserved and conserved mutations in positions “*a*” and “*d*” of HR1, nonconserved and conserved mutations in position “*a*” of HR2 and conserved mutations in position “*a*” of HR3 could inhibit fusion activity, except residue 289 mutation [32–36]. Compared to those of the other genotype XII NDVs, there were three variations in heptad repeats (residues 170 in HR1, 496 in HR2, and 270 in HR2) in positions “*f*,” “*c*,” and “*b*,” respectively. The role of these three nonconserved variations remains unknown, although a single mutation in the “*b*” position did not significantly disrupt the fusogenic ability of the F protein [33]. Signal peptides are located at the N-terminus of the F protein, marking the protein secretion pathway and the protein target location. It plays a role in controlling the rate of protein secretion, determining the protein folding state, affecting downstream transmembrane behavior, N-terminal glycosylation, and nuclear localization signal functionality and viral infectivity [37]; thus, the impact of nonconserved variation at residue 28 is unknown. The cytoplasmic tail is located at residues 523–553, which are the C-terminal residues of protein F. The mutation M553A resulted in increased (32% higher) fusion indices and viral titers in DF-1 cells in a previous study [38]; therefore, whether the variation A553V from subtypes XIIa and XIId to XIIb NDVs would impact pathogenicity and replication also needs to be further studied.

HN is a multifunctional protein and consists of four regions: the cytoplasmic tail, transmembrane domain, stalk regions, and globular head. The cytoplasmic tail is critical for replication, and the species-specific phenotypes based on only the homologous cytoplasmic tails of F and HN contained in chimeric NDVs could be recovered [39]. The mutation S6A in the cytoplasmic tail can affect cell fusion and colocalization of protein HN and M in cells [18]. On the other hand, two subtype XIIa NDVs had the nonconserved substitution S6D, which impaired viral assembly and replication in a chimeric genotype XII NDV vaccine [40], while the role in four subtype XIIb NDVs with the conserved variation S6N remains unknown. Variations at residue 2 may not have any impact, as a previous report described that deleting the first 2 amino acids did not affect the biological characteristics of NDV [18]. The HR motif in the transmembrane domain of protein HN has three conserved leucine residues (residues 30, 37, and 44 in position “*a*” in the heptad-repeat), and mutation of these residues impairs the attachment and fusion promotion activity of the protein HN [19]. The stalk region of the protein HN interacts with homologous F proteins to mediate the fusion process and is critical to replication [39, 41–43]. Mutations in the intervening region of the stalk region (89 to 95) or in two HRs of the stalk region (74, 81, 88, 90, 96, 97, 102, 103, 110, or combination mutation) variously decrease fusion promotion and HN-F interaction [41, 44]. Yan et al. [43] characterized L110 and R116 as playing a key role in determining the difference in virulence between two strains with 99.9% similarity. Five variations in the transmembrane domain and two variations in the stalk region were found between subtype XIIa and XIIb NDVs, and none of them were included in the residues that have been studied before. Two sialic acid-binding sites located in the globular head that play roles in binding to sialic acid receptors, removing sialic acid from newly synthesized viral coat proteins to prevent the aggregation of new viruses, and activating fusion by the protein F [22, 23, 45]. Mutation of these sialic acid-binding sites impaired NA and/or HAd activities. These two conserved residues of sialic acid-binding sites were conserved among all analyzed genotype XII NDVs in this study. In addition to two sialic acid-binding sites, mutations in residues 158, 160, 216, 220, 224, 232, 280, 289, 332, 336, 384, 495, 518, 536, and 557 reduced the fusion promotion activity of NDV [46]. Variations in residues 232 and 280 were found in this study. Only four (119, 341, 433, and 481) of six potential N-linked glycosylation sites used for carbohydrate addition were involved in cell attachment and fusion promotion activities of the protein in a previous report [47]; thus, variation of sites 508 and 538 may not influence the bioactivities of HN.

Protein V contributes to virus virulence via its IFN antagonistic activity [48]. One report on LaSota and Beaudette C showed that variation at residues 144, 153, 161, and 234 contributed to IFN antagonistic activity, and residues 30, 41, 46, 65, and 82 at the N-terminal region may also contribute to IFN antagonistic activity by affecting the structure of the protein, making the *C*-terminus less accessible to interacting host proteins to affectthe structure of the protein [49]. In this study, residue 161 of subtype XIIb NDVs was an *S*, consistent with that of LaSota, whereas it was a *P* in subtype XIIa NDVs, consistent with Beaudette C. Residue 65 of subtype XIIb NDVs was an *N*, as also observed in LaSota, whereas it was an S in subtype XIIa NDVs, but this variation did not change the properties of the amino acids.

By exchanging genes between velogenic strain Herts and lentogenic strain AV324A, it was observed that the correlation between virulence and the efficiency of viral replication in the viral replication complex (NP, P, and L) of NDVs [50]. Nuclear localization of protein M could also impact the virulence of NDVs through inhibiting host cell transcription, and promoting viral replication by affecting viral RNA synthesis and transcription [51]. Residues 40, 17, 25, and 15 variations were found in the P, NP, L, and M proteins.

In this study, numerous variations were found in six proteins between subtype XIIa and XIIb NDVs. Some of them have been studied in cells to determine their roles in previous studies, yet their role in animals still needs to be researched. Considering the pathogenicity differences in chickens via the natural route, some regions must play a role in this phenomenon, which remains unknown, and the variations among genotype XII NDVs would be suitable materials.

Genotype XII NDVs have been reported in chickens, gamecocks, geese, and peacocks, while they were isolated from wild birds for the first time in this study, as subtype XIIb NDVs were previously isolated only in geese. Poultry-derived NDVs have been proven to spill over to wild birds [52], especially migratory birds, and spillover events from wild birds to poultry also exist [53]. Francolins are common resident birds in southern China that live around poultry farms where the poultry are fed on the open ground, which is a popular breeding method in southern China. Unlike migratory birds, resident birds often stay in the poultry farm, e.g., eating poultry feed and getting in close contact with poultry. It would facilitate infectious disease spread to each other. Subtype XIIb NDVs have only been found in Guangdong and Guangxi provinces to date, and it seems that resident birds play a more major role than migratory birds in spreading subtype XIIb NDVs over short distances to form an endemic situation. According to the phylogenetic tree, two francolins-derived NDVs were divided into a branch with an NDV isolated in Guangdong in 2013, whereas two goose-derived NDVs in Guangxi in 2018 with E115 in Guangdong in 2017 were divided into another branch, and none of the NDVs in this genotype were monitored between 2014 and 2017 under the continuous surveillance program in poultry, indicating that subtype XIIb NDVs have multiple transmission chains and that resident birds may be involved in this branch as intermediate hosts in which the virus also continues to evolve. In summary, it reminds us that infectious disease surveillance should be conducted in both migratory and resident birds, and subtype XIId NDVs in Vietnam could be a potential risk to poultry in China because Guangxi Province shares a long border with Vietnam.

The protective efficacy of subtype XIIb NDVs as inactivated vaccines was evaluated in chickens in this study. However, according to the statistical analysis results for viral shedding, there were no significant differences among the three vaccine-treated groups at 3, 5, and 7 dpi for both routes, and viral shedding could not be detected in the GX01-immunized group from 5 dpi for both routes, whereas it could be detected at 5 dpi in the LaSota and the A-VII-immunized group for the oropharyngeal route, and was detected at 5 dpi in the LaSota-immunized group for the cloacal route. This demonstrated that the GX01 vaccine could eliminate viral shedding faster. These results were similar to those showing the protective efficacy of a subtype XIIa chimeric vaccine against the homologous virus to a certain degree [40].

The chickens with the HI titer at 10 log_2_ were chosen for the inoculation with GX01 to eliminate the effects due to inconsistent antibody levels, and the results support the theory that a genotype-matched vaccine with similar antigen epitopes was more efficient than the serum antibody enrichment as an immunologic mechanism that still needs to be elucidated, with two major possibilities suggested by previous studies: local immunity such as mucosal immunity was increased and more specific with similar antigen epitopes in vaccine; cellular immunity was increased to clear the infection faster. Based on the result, subtype XIIb NDVs could be a candidate vaccine strain against subtype XIId NDVs on account of the potential risk of spreading to China and increased pathogenicity of Subtype XIIb NDVs.

In conclusion, two francolin-derived subtype XIIb NDV strains, GX01 and GX02 were isolated and characterized as velogenic NDVs in Guangxi, southwestern China. Based on the weaker pathogenicity in chickens, 17, 40, 15, 7, 32, 25, and 31 amino acid variations were found in proteins NP, P, M, F, HN, L, and V, and some of these variations may be responsible for this phenomenon. Based on epidemiology and phylogenetic analyses, we were reminded that infectious disease surveillance should be conducted in both migratory and resident birds. Because of the potential risk of subtype XIId NDVs spreading to China and the increased pathogenicity of subtype XIIb NDVs, the protective efficacy of GX01 as an inactivated vaccine was evaluated with two commercial inactivated vaccines in chickens. The results showed that subtype XIIb NDVs could be candidate genotype-matched vaccine strains against genotype XII NDVs.

## Figures and Tables

**Table 1:** Statistical analysis of viral shedding in different vaccine-immunized groups.

Days post inoculation	Group					
	Vaccinated with XII-GX01/vaccinated with II-LaSota	Vaccinated with XII-GX01/vaccinated with VII-A-VII	Vaccinated with XII-GX01/nonvaccinated	Vaccinated with II-LaSota/vaccinated with VII-A-VII	Vaccinated with II-LaSota/nonvaccinated	Vaccinated with VII-A-VII/nonvaccinated
Cloacal						
1	n.s.	n.s.	^*∗*^	n.s.	n.s.	n.s.
3	n.s.	n.s.	^*∗∗∗*^	n.s.	^*∗∗∗*^	^*∗∗∗*^
5	n.s.	n.s.	^*∗∗∗*^	n.s.	^*∗∗∗*^	^*∗∗∗*^
7	n.s.	n.s.	^*∗∗∗*^	n.s.	^*∗∗∗*^	^*∗∗∗*^
Oropharyngeal						
1	n.s.	n.s.	^*∗*^	n.s.	n.s.	n.s.
3	n.s.	n.s.	^*∗∗∗*^	n.s.	^*∗∗∗*^	^*∗∗∗*^
5	n.s.	n.s.	^*∗∗∗*^	n.s.	^*∗∗∗*^	^*∗∗∗*^
7	n.s.	n.s.	^*∗∗∗*^	n.s.	^*∗∗∗*^	^*∗∗∗*^

n.s. *P*  >  0.05, not significantly different. ^*∗*^0.01 < *P* < 0.05, ^*∗∗*^0.001 < *P* < 0.01, and ^*∗∗∗*^ *P* < 0.001.

## Data Availability

The datasets generated and analyzed during the current study are available from the corresponding author upon reasonable request.

## Supplementary Materials

Supplementary Materials Table 1: variations in protein F. Table 2: variations in protein HN. Table 3: variations in the NP and M proteins. Table 4: variations in protein L. Table 5: variations in protein L. Table 6: variations in protein P. Table 7: variations in protein V. Table 8: variations in the neutralizing epitopes of proteins F and HN. Table 9: variations between only francolin strains and other genotype XII NDVs. Table 10: the EID_50_ values from cloacal swabs (log_10_).Table 11: the EID_50_ values from oropharyngeal swabs (log_10_).
