# Supplementary material for: Phylogeny and Pathogenicity of Subtype XIIb NDVs from Francolins in Southwestern China and Effective Protection by an Inactivated Vaccine
Source: Transbound Emerg Dis. 2023 Apr 5;2023:1317784. doi: 10.1155/2023/1317784 (PMC12017135; doi:10.1155/2023/1317784)
Supplement: Supplementary Materials — Table 1: variations in protein F. Table 2: variations in protein HN. Table 3: variations in the NP and M proteins. Table 4: variations in protein L. Table 5: variations in protein L. Table 6: variations in protein P. Table 7: variations in protein V. Table 8: variations in the neutralizing epitopes of proteins F and HN. Table 9: variations between only francolin strains and other genotype XII NDVs. Table 10: the EID50 values from cloacal swabs (log10).Table 11: the EID50 values from oropharyngeal swabs (log10). [file 1317784.f1.zip › supplement tables10.docx]

**Table 10.** The EID_50_ values from cloacal swabs (log_10_)

|  | dpi1 | dpi3 | dpi5 | dpi7 | dpi9 | dpi11 | dpi13 | dpi15 |
| --- | --- | --- | --- | --- | --- | --- | --- | --- |
| Vaccine |  |  |  |  |  |  |  |  |
| Ⅻ- GX01 | 1.5 | 1.375 | 0 | 0 | 0 | 0 | 0 | 0 |
|  | 1.168 | 1.375 | 0 | 0 | 0 | 0 | 0 | 0 |
|  | 1.168 | 0 | 0 | 0 | 0 | 0 | 0 | 0 |
|  | 0 | 0 | 0 | 0 | 0 | 0 | 0 | 0 |
|  | 0 | 0 | 0 | 0 | 0 | 0 | 0 | 0 |
|  | 0 | 0 | 0 | 0 | 0 | 0 | 0 | 0 |
|  | 0 | 0 | 0 | 0 | 0 | 0 | 0 | 0 |
|  | 0 | 0 | 0 | 0 | 0 | 0 | 0 | 0 |
| Ⅱ - LaSota | 1.833 | 1.625 | 1.167 | 1.167 | 0 | 0 | 0 | 0 |
|  | 1.167 | 1.5 | 1.375 | 1.167 | 0 | 0 | 0 | 0 |
|  | 1.375 | 0 | 0 | 0 | 0 | 0 | 0 | 0 |
|  | 1.625 | 0 | 0 | 0 | 0 | 0 | 0 | 0 |
|  | 1.375 | 0 | 0 | 0 | 0 | 0 | 0 | 0 |
|  | 1.833 | 0 | 0 | 0 | 0 | 0 | 0 | 0 |
|  | 0 | 0 | 0 | 0 | 0 | 0 | 0 | 0 |
|  | 0 | 0 | 0 | 0 | 0 | 0 | 0 | 0 |
| Ⅶ- A-Ⅶ | 1.375 | 1.833 | 0 | 0 | 0 | 0 | 0 | 0 |
|  | 1.375 | 1.375 | 0 | 0 | 0 | 0 | 0 | 0 |
|  | 1.375 | 1.625 | 0 | 0 | 0 | 0 | 0 | 0 |
|  | 1.625 | 0 | 0 | 0 | 0 | 0 | 0 | 0 |
|  | 0 | 0 | 0 | 0 | 0 | 0 | 0 | 0 |
|  | 0 | 0 | 0 | 0 | 0 | 0 | 0 | 0 |
|  | 0 | 0 | 0 | 0 | 0 | 0 | 0 | 0 |
|  | 0 | 0 | 0 | 0 | 0 | 0 | 0 | 0 |
| PBS | 1.833 | 4.375 | 2.375 | 2.375 | 1.375 | 1.375 | 0 | 0 |
|  | 1.375 | 4.375 | 2.5 | 2.375 | 1.167 | 1.167 | 0 | 0 |
|  | 1.5 | 4.375 | 2.5 | 2.5 | 1.833 | 1.167 | 0 | 0 |
|  | 1.833 | 4.5 | 2.833 | 2.375 | 1.375 | 0 | 0 | 0 |
|  | 1.5 | 4.5 | 2.681 | 2.375 | 0 | 0 | 0 | 0 |
|  | 1.375 | 4.375 | 2.681 | 2.375 | 0 | 0 | 0 | 0 |
|  | 1.833 | 4.375 | 2.375 | 2.375 | 0 | 0 | 0 | 0 |
|  | 0 | 4.375 | 2.375 | 2.375 | 0 | 0 | 0 | 0 |
