# Supplementary material for: Phylogeny and Pathogenicity of Subtype XIIb NDVs from Francolins in Southwestern China and Effective Protection by an Inactivated Vaccine
Source: Transbound Emerg Dis. 2023 Apr 5;2023:1317784. doi: 10.1155/2023/1317784 (PMC12017135; doi:10.1155/2023/1317784)
Supplement: Supplementary Materials — Table 1: variations in protein F. Table 2: variations in protein HN. Table 3: variations in the NP and M proteins. Table 4: variations in protein L. Table 5: variations in protein L. Table 6: variations in protein P. Table 7: variations in protein V. Table 8: variations in the neutralizing epitopes of proteins F and HN. Table 9: variations between only francolin strains and other genotype XII NDVs. Table 10: the EID50 values from cloacal swabs (log10).Table 11: the EID50 values from oropharyngeal swabs (log10). [file 1317784.f1.zip › supplement tables2.docx]

**Table 2.** Variations in protein HN

| Virus | HN | | | | | | | | | | | | | | | | | | | | | | | | | | | | | | | |
| --- | --- | --- | --- | --- | --- | --- | --- | --- | --- | --- | --- | --- | --- | --- | --- | --- | --- | --- | --- | --- | --- | --- | --- | --- | --- | --- | --- | --- | --- | --- | --- | --- |
|  | Cytoplasmic tail  (1-26)^a^ | | | | | Transmembrane domain  (27-45) | | | | | Stalk region  (49-123) | | Globular head (124-571) | | | | | | | | | | | | | | | | | | | |
|  | 2^a^ | 6 | 7 | 15 | 24 | 27 | 28 | 34 | 41 | 42 | 58 | 72 | 149 | 150 | 232 | 263 | 280 | 282 | 284 | 309 | 347 | 373 | 404 | 430 | 432 | 443 | 457 | 479 | 480 | 494 | 508 | 568 |
| Subtype Ⅻb (isolates in China) |  |  |  |  |  |  |  |  |  |  |  |  |  |  |  |  |  |  |  |  |  |  |  |  |  |  |  |  |  |  |  |  |
| MZ306226  francolin/China/GX01/2017 | N | N | R | K | I | I | A | I | T | A | G | A | I | S | T | R | Q | H | R | N | D | I | I | A | S | I | A | H | K | D | N | N |
| MZ306225  francolin/China/GX02/2017 | N | N | R | K | I | I | A | I | T | A | G | A | I | S | T | R | Q | H | R | N | D | I | I | A | S | I | A | H | K | D | N | N |
| MZ306224  Goose/China/GX02/2018 | N | S | R | K | I | I | A | I | T | A | G | A | I | S | T | R | Q | H | R | N | D | I | I | A | S | I | A | H | K | D | N | N |
| MZ306223  Goose/China/GX17/2018 | N | S | R | K | I | I | A | I | T | A | G | A | I | S | T | R | Q | H | R | N | D | I | I | A | S | I | A | H | K | D | N | N |
| MK616244  Goose/CH/GD/E115/2017 | N | N | R | K | I | I | A | I | T | A | G | A | I | S | T | R | Q | H | R | N | D | I | I | A | S | I | A | H | K | D | N | N |
| KC551967  Goose/Guangdong/2010 | N | N | R | K | I | I | A | I | T | A | G | A | T | S | T | R | Q | H | R | N | D | I | I | A | S | I | A | H | K | D | N | N |
| Subtype Ⅻa (isolates in South America) |  |  |  |  |  |  |  |  |  |  |  |  |  |  |  |  |  |  |  |  |  |  |  |  |  |  |  |  |  |  |  |  |
| JN800306  Chicken/Peru/1918-03/603/2008 | G | D | K | R | V | V | S | V | A | V | N | S | T | L | S | K | R | R | K | D | E | V | V | T | D | T | S | Y | R | G | S | D |
| KR732614  NDV/peacock/Peru/2011 | G | D | K | R | V | V | S | V | A | V | N | S | T | L | S | K | R | R | K | D | E | V | V | T | D | T | S | Y | R | G | S | D |

Note: ^a^ The numbers at the bottom of the column headings in the tables indicate the amino acid numbering.
