# Supplementary material for: Phylogeny and Pathogenicity of Subtype XIIb NDVs from Francolins in Southwestern China and Effective Protection by an Inactivated Vaccine
Source: Transbound Emerg Dis. 2023 Apr 5;2023:1317784. doi: 10.1155/2023/1317784 (PMC12017135; doi:10.1155/2023/1317784)
Supplement: Supplementary Materials — Table 1: variations in protein F. Table 2: variations in protein HN. Table 3: variations in the NP and M proteins. Table 4: variations in protein L. Table 5: variations in protein L. Table 6: variations in protein P. Table 7: variations in protein V. Table 8: variations in the neutralizing epitopes of proteins F and HN. Table 9: variations between only francolin strains and other genotype XII NDVs. Table 10: the EID50 values from cloacal swabs (log10).Table 11: the EID50 values from oropharyngeal swabs (log10). [file 1317784.f1.zip › supplement tables3.docx]

**Table 3.** Variations in the NP and M proteins

| Virus | NP | | | | | | | | | | | | | | | | | M | | | | | | | | | | | | | | |
| --- | --- | --- | --- | --- | --- | --- | --- | --- | --- | --- | --- | --- | --- | --- | --- | --- | --- | --- | --- | --- | --- | --- | --- | --- | --- | --- | --- | --- | --- | --- | --- | --- |
|  | 22^a^ | 35 | 93 | 100 | 108 | 123 | 208 | 321 | 403 | 408 | 426 | 427 | 433 | 434 | 467 | 470 | 472 | 26 | 83 | 84 | 98 | 100 | 115 | 116 | 221 | 247 | 248 | 250 | 323 | 347 | 349 | 353 |
| Subtype Ⅻb (isolates in China) |  |  |  |  |  |  |  |  |  |  |  |  |  |  |  |  |  |  |  |  |  |  |  |  |  |  |  |  |  |  |  |  |
| MZ306226 francolin/China/GX01/2017 | Q | I | K | A | T | E | V | N | I | V | V | S | R | P | S | L | E | V | K | R | L | N | V | T | I | K | R | K | Q | T | K | R |
| MZ306225  francolin/China/GX02/2017 | Q | I | K | A | T | E | V | N | I | V | V | S | R | P | S | L | E | V | K | R | L | N | V | T | I | K | R | K | Q | T | K | R |
| MZ306224  Goose/China/GX02/2018 | Q | I | K | A | T | E | V | N | I | V | V | S | R | P | S | L | E | V | K | R | L | N | V | T | I | K | R | K | Q | T | K | R |
| MZ306223  Goose/China/GX17/2018 | Q | I | K | A | T | E | V | N | I | V | V | S | R | P | S | L | E | V | K | R | L | N | V | T | I | K | R | K | Q | T | K | R |
| MK616244  Goose/CH/GD/E115/2017 | Q | I | K | A | T | E | V | N | I | V | V | S | R | P | S | L | E | V | K | R | L | N | V | T | I | K | R | K | Q | T | K | R |
| KC551967  Goose/Guangdong/2010 | Q | I | K | A | T | E | V | N | I | V | V | S | R | P | S | L | E | V | K | R | L | N | V | T | I | K | R | K | Q | T | K | R |
| Subtype Ⅻa (isolates in South America) |  |  |  |  |  |  |  |  |  |  |  |  |  |  |  |  |  |  |  |  |  |  |  |  |  |  |  |  |  |  |  |  |
| JN800306  Chicken/Peru/1918-03/603/2008 | H | V | R | V | A | D | I | S | T | I | A | P | K | T | G | P | D | I | R | H | P | D | I | N | V | R | K | R | R | S | R | K |
| KR732614  NDV/peacock/Peru/2011 | H | V | R | V | A | D | I | S | T | I | A | P | K | T | G | P | D | I | R | H | P | D | I | N | V | R | K | R | R | S | R | K |

Note: ^a^ The numbers at the bottom of the column headings in the tables indicate the amino acid numbering.
