# Supplementary material for: Phylogeny and Pathogenicity of Subtype XIIb NDVs from Francolins in Southwestern China and Effective Protection by an Inactivated Vaccine
Source: Transbound Emerg Dis. 2023 Apr 5;2023:1317784. doi: 10.1155/2023/1317784 (PMC12017135; doi:10.1155/2023/1317784)
Supplement: Supplementary Materials — Table 1: variations in protein F. Table 2: variations in protein HN. Table 3: variations in the NP and M proteins. Table 4: variations in protein L. Table 5: variations in protein L. Table 6: variations in protein P. Table 7: variations in protein V. Table 8: variations in the neutralizing epitopes of proteins F and HN. Table 9: variations between only francolin strains and other genotype XII NDVs. Table 10: the EID50 values from cloacal swabs (log10).Table 11: the EID50 values from oropharyngeal swabs (log10). [file 1317784.f1.zip › supplement tables4.docx]

**Table 4.** Variations in protein L

| Virus | L | | | | | | | | | | | | | | | | | | | | | | | | |
| --- | --- | --- | --- | --- | --- | --- | --- | --- | --- | --- | --- | --- | --- | --- | --- | --- | --- | --- | --- | --- | --- | --- | --- | --- | --- |
|  | 45^a^ | 46 | 62 | 104 | 149 | 172 | 206 | 208 | 221 | 241 | 258 | 271 | 324 | 329 | 331 | 334 | 369 | 372 | 425 | 497 | 759 | 853 | 889 | 895 | 897 |
| Subtype Ⅻb (isolates in China) |  |  |  |  |  |  |  |  |  |  |  |  |  |  |  |  |  |  |  |  |  |  |  |  |  |
| MZ306226 francolin/China/GX01/2017 | E | Y | T | S | P | A | T | Q | I | I | K | I | K | N | A | V | N | R | I | V | R | I | S | S | P |
| MZ306225  francolin/China/GX02/2017 | E | Y | T | S | P | A | T | Q | I | I | K | I | K | N | A | V | N | R | I | V | R | I | S | S | P |
| MZ306224  Goose/China/GX02/2018 | E | Y | T | S | P | A | T | Q | I | I | K | I | K | N | A | V | N | R | I | V | R | I | S | S | P |
| MZ306223  Goose/China/GX17/2018 | E | Y | T | S | P | A | T | Q | I | I | K | I | K | N | A | V | N | R | I | V | R | I | S | S | P |
| MK616244  Goose/CH/GD/E115/2017 | E | Y | T | S | P | A | T | Q | I | I | K | I | K | N | A | V | N | R | I | V | R | I | S | S | P |
| KC551967  Goose/Guangdong/2010 | E | Y | T | S | P | A | T | Q | I | I | K | I | K | N | A | V | N | R | I | V | R | I | S | S | P |
| Subtype Ⅻa (isolates in South America) |  |  |  |  |  |  |  |  |  |  |  |  |  |  |  |  |  |  |  |  |  |  |  |  |  |
| JN800306  Chicken/Peru/1918-03/603/2008 | D | F | A | P | L | S | I | H | T | M | R | V | E | D | V | I | S | K | L | I | K | V | N | N | S |
| KR732614  NDV/peacock/Peru/2011 | D | F | A | P | L | S | I | H | T | M | R | V | E | D | V | I | S | K | L | I | K | V | N | N | S |

Note: ^a^ The numbers at the bottom of the column headings in the tables indicate the amino acid numbering.
