# Supplementary material for: Phylogeny and Pathogenicity of Subtype XIIb NDVs from Francolins in Southwestern China and Effective Protection by an Inactivated Vaccine
Source: Transbound Emerg Dis. 2023 Apr 5;2023:1317784. doi: 10.1155/2023/1317784 (PMC12017135; doi:10.1155/2023/1317784)
Supplement: Supplementary Materials — Table 1: variations in protein F. Table 2: variations in protein HN. Table 3: variations in the NP and M proteins. Table 4: variations in protein L. Table 5: variations in protein L. Table 6: variations in protein P. Table 7: variations in protein V. Table 8: variations in the neutralizing epitopes of proteins F and HN. Table 9: variations between only francolin strains and other genotype XII NDVs. Table 10: the EID50 values from cloacal swabs (log10).Table 11: the EID50 values from oropharyngeal swabs (log10). [file 1317784.f1.zip › supplement tables5.docx]

**Table 5.** Variations in protein L

| Virus | L | | | | | | | | | | | | | | | | | | | | | | | | | | | | |
| --- | --- | --- | --- | --- | --- | --- | --- | --- | --- | --- | --- | --- | --- | --- | --- | --- | --- | --- | --- | --- | --- | --- | --- | --- | --- | --- | --- | --- | --- |
|  | 1110^a^ | 1122 | 1133 | 1184 | 1363 | 1561 | 1633 | 1684 | 1687 | 1712 | 1722 | 1734 | 1738 | 1770 | 1792 | 1833 | 1851 | 1864 | 1939 | 1958 | 1963 | 1971 | 2050 | 2055 | 2057 | 2058 | 2062 | 2094 | 2201 |
| Subtype Ⅻb (isolates in China) |  |  |  |  |  |  |  |  |  |  |  |  |  |  |  |  |  |  |  |  |  |  |  |  |  |  |  |  |  |
| MZ306226  francolin/China/GX01/2017 | N | S | K | R | I | F | Y | P | V | S | I | A | Y | K | I | E | I | S | R | I | Y | H | L | R | T | Q | N | T | T |
| MZ306225  francolin/China/GX02/2017 | N | S | K | R | I | F | Y | P | V | S | I | A | Y | K | I | E | I | S | R | I | Y | H | L | R | T | Q | N | T | T |
| MZ306224  Goose/China/GX02/2018 | N | S | K | R | I | F | Y | P | V | S | I | A | Y | K | I | E | I | S | R | I | Y | H | L | R | T | Q | N | T | T |
| MZ306223  Goose/China/GX17/2018 | N | S | K | R | I | F | Y | P | V | S | I | A | Y | K | I | E | I | S | R | I | Y | H | L | R | T | Q | N | T | T |
| MK616244  Goose/CH/GD/E115/2017 | N | S | K | R | I | F | Y | P | V | S | I | A | Y | K | I | E | I | S | R | I | Y | H | L | R | T | Q | N | T | T |
| KC551967  Goose/Guangdong/2010 | N | S | K | R | I | F | Y | P | V | S | I | A | Y | K | I | E | I | S | R | I | Y | H | L | R | T | Q | N | T | T |
| Subtype Ⅻa (isolates in South America) |  |  |  |  |  |  |  |  |  |  |  |  |  |  |  |  |  |  |  |  |  |  |  |  |  |  |  |  |  |
| JN800306  Chicken/Peru/1918-03/603/2008 | D | N | R | K | L | C | S | I | I | C | L | P | H | R | V | G | N | G | K | V | C | Q | I | Q | N | R | S | I | N |
| KR732614  NDV/peacock/Peru/2011 | D | N | R | K | L | C | S | I | I | C | L | P | H | R | V | G | N | G | K | V | C | Q | I | Q | N | R | S | I | N |

Note: ^a^ The numbers at the bottom of the column headings in the tables indicate the amino acid numbering.
