# Supplementary material for: Phylogeny and Pathogenicity of Subtype XIIb NDVs from Francolins in Southwestern China and Effective Protection by an Inactivated Vaccine
Source: Transbound Emerg Dis. 2023 Apr 5;2023:1317784. doi: 10.1155/2023/1317784 (PMC12017135; doi:10.1155/2023/1317784)
Supplement: Supplementary Materials — Table 1: variations in protein F. Table 2: variations in protein HN. Table 3: variations in the NP and M proteins. Table 4: variations in protein L. Table 5: variations in protein L. Table 6: variations in protein P. Table 7: variations in protein V. Table 8: variations in the neutralizing epitopes of proteins F and HN. Table 9: variations between only francolin strains and other genotype XII NDVs. Table 10: the EID50 values from cloacal swabs (log10).Table 11: the EID50 values from oropharyngeal swabs (log10). [file 1317784.f1.zip › supplement tables6.docx]

**Table 6.** Variations in protein P

| Virus | P | | | | | | | | | | | | | | | | | | | | | | | | | | | | | | | | | | | | | | | |
| --- | --- | --- | --- | --- | --- | --- | --- | --- | --- | --- | --- | --- | --- | --- | --- | --- | --- | --- | --- | --- | --- | --- | --- | --- | --- | --- | --- | --- | --- | --- | --- | --- | --- | --- | --- | --- | --- | --- | --- | --- |
|  | 38^a^ | 39 | 45 | 47 | 55 | 58 | 61 | 65 | 75 | 76 | 89 | 103 | 136 | 146 | 147 | 154 | 155 | 169 | 170 | 172 | 174 | 187 | 202 | 203 | 206 | 210 | 213 | 230 | 238 | 241 | 322 | 323 | 331 | 334 | 341 | 342 | 343 | 344 | 380 | 383 |
| Subtype Ⅻb (isolates in China) |  |  |  |  |  |  |  |  |  |  |  |  |  |  |  |  |  |  |  |  |  |  |  |  |  |  |  |  |  |  |  |  |  |  |  |  |  |  |  |  |
| MZ306226 francolin/China/GX01/2017 | T | T | R | P | E | R | V | N | H | S | T | G | N | S | P | R | L | K | A | P | N | R | R | L | S | A | P | M | V | V | L | T | V | P | A | T | A | C | M | E |
| MZ306225  francolin/China/GX02/2017 | T | T | R | P | E | R | V | N | H | S | T | G | N | S | P | R | L | K | A | P | N | R | R | L | S | A | P | M | V | V | L | T | V | P | A | T | A | C | M | E |
| MZ306224  Goose/China/GX02/2018 | T | T | R | P | E | R | V | N | H | S | T | G | N | S | P | R | L | K | A | P | N | R | R | L | S | A | P | M | V | V | L | T | V | P | A | T | A | C | M | E |
| MZ306223  Goose/China/GX17/2018 | T | T | R | P | E | R | V | N | H | S | T | G | N | S | P | R | L | K | A | P | N | R | R | L | S | A | P | M | V | V | L | T | V | P | A | T | A | C | M | E |
| MK616244  Goose/CH/GD/E115/2017 | T | T | R | P | E | R | V | N | H | S | T | G | N | S | P | R | L | K | A | P | N | R | R | L | S | A | P | M | V | V | L | T | V | P | A | T | A | C | M | E |
| KC551967  Goose/Guangdong/2010 | T | T | R | P | E | R | V | N | H | S | T | G | N | S | P | R | L | K | A | P | N | R | R | L | S | A | P | M | V | V | L | T | V | P | A | T | A | C | I | E |
| Subtype Ⅻa (isolates in South America) |  |  |  |  |  |  |  |  |  |  |  |  |  |  |  |  |  |  |  |  |  |  |  |  |  |  |  |  |  |  |  |  |  |  |  |  |  |  |  |  |
| JN800306  Chicken/Peru/1918-03/603/2008 | A | I | K | L | G | Q | S | S | Q | P | P | E | R | P | L | Q | P | G | I | S | S | Q | P | S | N | T | S | T | A | I | M | A | I | H | T | A | V | S | R | D |
| KR732614  NDV/peacock/Peru/2011 | A | I | K | L | G | Q | S | S | Q | P | P | E | R | P | L | Q | P | G | I | S | S | Q | P | S | N | T | S | T | A | I | M | A | I | H | T | A | V | S | R | D |

Note: ^a^ The numbers at the bottom of the column headings in the tables indicate the amino acid numbering.
