# Supplementary material for: Phylogeny and Pathogenicity of Subtype XIIb NDVs from Francolins in Southwestern China and Effective Protection by an Inactivated Vaccine
Source: Transbound Emerg Dis. 2023 Apr 5;2023:1317784. doi: 10.1155/2023/1317784 (PMC12017135; doi:10.1155/2023/1317784)
Supplement: Supplementary Materials — Table 1: variations in protein F. Table 2: variations in protein HN. Table 3: variations in the NP and M proteins. Table 4: variations in protein L. Table 5: variations in protein L. Table 6: variations in protein P. Table 7: variations in protein V. Table 8: variations in the neutralizing epitopes of proteins F and HN. Table 9: variations between only francolin strains and other genotype XII NDVs. Table 10: the EID50 values from cloacal swabs (log10).Table 11: the EID50 values from oropharyngeal swabs (log10). [file 1317784.f1.zip › supplement tables7.docx]

**Table 7.** Variations in protein V

| Virus | V | | | | | | | | | | | | | | | | | | | | | | | | | | | | | | |
| --- | --- | --- | --- | --- | --- | --- | --- | --- | --- | --- | --- | --- | --- | --- | --- | --- | --- | --- | --- | --- | --- | --- | --- | --- | --- | --- | --- | --- | --- | --- | --- |
|  | 38^a^ | 39 | 45 | 47 | 55 | 58 | 61 | 65 | 75 | 76 | 89 | 103 | 136 | 137 | 140 | 142 | 146 | 152 | 157 | 159 | 161 | 162 | 168 | 169 | 170 | 172 | 203 | 210 | 213 | 215 | 239 |
| Subtype Ⅻb (isolates in China) |  |  |  |  |  |  |  |  |  |  |  |  |  |  |  |  |  |  |  |  |  |  |  |  |  |  |  |  |  |  |  |
| MZ306226 francolin/China/GX01/2017 | T | T | R | P | E | R | V | N | H | S | T | G | E | Y | P | G | I | R | S | K | S | G | G | Q | G | P | V | C | T | G | K |
| MZ306225  francolin/China/GX02/2017 | T | T | R | P | E | R | V | N | H | S | T | G | E | Y | P | G | I | R | S | K | S | G | G | Q | G | P | V | C | T | G | K |
| MZ306224  goose/China/GX02/2018 | T | T | R | P | E | R | V | N | H | S | T | G | E | Y | P | G | I | R | S | K | S | G | G | Q | G | P | V | C | T | G | K |
| MZ306223  goose/China/GX17/2018 | T | T | R | P | E | R | V | N | H | S | T | G | E | Y | P | G | I | R | S | K | S | G | G | Q | G | P | V | C | T | G | K |
| MK616244  goose/CH/GD/E115/2017 | T | T | R | P | E | R | V | N | H | S | T | G | E | Y | P | G | I | R | S | K | S | G | G | Q | G | P | V | C | T | G | K |
| KC551967  goose/Guangdong/2010 | T | T | R | P | E | R | V | N | H | S | T | G | E | Y | P | G | I | R | S | K | S | G | G | Q | G | P | V | C | T | G | K |
| Subtype Ⅻa (isolates in South America) |  |  |  |  |  |  |  |  |  |  |  |  |  |  |  |  |  |  |  |  |  |  |  |  |  |  |  |  |  |  |  |
| JN800306  chicken/Peru/1918-03/603/2008 | A | I | K | L | G | Q | S | S | Q | P | P | E | K | D | S | R | T | Q | P | E | P | R | S | R | D | L | I | Y | I | R | E |
| KR732614  NDV/peacock/Peru/2011 | A | I | K | L | G | Q | S | S | Q | P | P | E | K | D | S | R | T | Q | P | E | P | R | S | R | D | L | I | Y | I | R | E |

Note: ^a^ The numbers at the bottom of the column headings in the tables indicate the amino acid numbering.
