# Supplementary material for: Phylogeny and Pathogenicity of Subtype XIIb NDVs from Francolins in Southwestern China and Effective Protection by an Inactivated Vaccine
Source: Transbound Emerg Dis. 2023 Apr 5;2023:1317784. doi: 10.1155/2023/1317784 (PMC12017135; doi:10.1155/2023/1317784)
Supplement: Supplementary Materials — Table 1: variations in protein F. Table 2: variations in protein HN. Table 3: variations in the NP and M proteins. Table 4: variations in protein L. Table 5: variations in protein L. Table 6: variations in protein P. Table 7: variations in protein V. Table 8: variations in the neutralizing epitopes of proteins F and HN. Table 9: variations between only francolin strains and other genotype XII NDVs. Table 10: the EID50 values from cloacal swabs (log10).Table 11: the EID50 values from oropharyngeal swabs (log10). [file 1317784.f1.zip › supplement tables8.docx]

**Table 8.** Variations in the neutralizing epitopes of proteins F and HN

|  | F | | | | | | | HN | | | | | | |
| --- | --- | --- | --- | --- | --- | --- | --- | --- | --- | --- | --- | --- | --- | --- |
|  | 72^a^ | 74 | 75 | 78 | 79 | 157-171 | 343 | 345 | 513,514,  521,569 | 236,287,321, | 332,333,356 | 494,516 | 347,350,353 | 193,194,201 |
| Commercial vaccine |  |  |  |  |  |  |  |  |  |  |  |  |  |  |
| LaSota | D | E | A | K | A | SIAATNEAVHEVTDG | L | P | R, I, S, D | K, D, K | G, K, K | G, R | E, Y, R | L, S, H |
| A-Ⅶ | D | E | A | K | A | SIAATNEAVHEVTDG | L | P | R, V, S, D | K, D, K | G, K, K | D, R | E, Y, R | L, S, H |
| Subtype XIIb (isolates in China) |  |  |  |  |  |  |  |  |  |  |  |  |  |  |
| MZ306226 francolin/China/GX01/2017 | D | E | A | K | A | SIAATNEAVHEVTNG | L | P | R, V, N, D | K, D, K | G, K, K | D, R | D, Y, R | L, S, H |
| MZ306225 francolin/China/GX02/2017 | D | E | A | K | A | SIAATNEAVHEVTNG | L | P | R, V, N, D | K, D, K | G, K, K | D, R | D, Y, R | L, S, H |
| MZ306224  Goose/China/GX02/2018 | D | E | A | K | A | SIAATNEAVHEVTNG | L | P | R, V, N, D | K, D, K | G, K, K | D, R | D, Y, R | L, S, H |
| MZ306223  Goose/China/GX17/2018 | D | E | A | K | A | SIAATNEAVHEVTNG | L | P | R, V, N, D | K, D, K | G, K, K | D, R | D, Y, R | L, S, H |
| MK616244  Goose/CH/GD/E115/2017 | D | E | A | K | A | SIAATNEAVHEVTNG | L | P | R, V, N, D | K, D, K | G, K, K | D, R | D, Y, R | L, S, H |
| JN627504  Goose/GD12/2011 | D | E | A | K | A | SIAATNEAVHEVTNG | L |  |  |  |  |  |  |  |
| JN627505  Goose/GD17/2011 | D | E | A | K | A | SIAATNEAVHEVTNG | L |  |  |  |  |  |  |  |
| JN627506  Goose/GD20/2011 | D | E | A | K | A | SIAATNEAVHEVTNG | L |  |  |  |  |  |  |  |
| JN627507  Goose/GD1003/2010 | D | E | A | K | A | SIAATNEAVHEVTNG | L |  |  |  |  |  |  |  |
| JN627508  Goose/GD450/2011 | D | E | A | K | A | SIAATNEAVHEVTNG | L |  |  |  |  |  |  |  |
| KC551967  Goose/Guangdong/2010 | D | E | A | K | A | SIAATNEAVHEVTNG | L | P | R, V, N, D | K, D, K | G, K, K | D, R | D, Y, R | L, S, H |

Note: ^a^ The numbers at the bottom of the column headings in the tables indicate the amino acid numbering.
