# Supplementary material for: Phylogeny and Pathogenicity of Subtype XIIb NDVs from Francolins in Southwestern China and Effective Protection by an Inactivated Vaccine
Source: Transbound Emerg Dis. 2023 Apr 5;2023:1317784. doi: 10.1155/2023/1317784 (PMC12017135; doi:10.1155/2023/1317784)
Supplement: Supplementary Materials — Table 1: variations in protein F. Table 2: variations in protein HN. Table 3: variations in the NP and M proteins. Table 4: variations in protein L. Table 5: variations in protein L. Table 6: variations in protein P. Table 7: variations in protein V. Table 8: variations in the neutralizing epitopes of proteins F and HN. Table 9: variations between only francolin strains and other genotype XII NDVs. Table 10: the EID50 values from cloacal swabs (log10).Table 11: the EID50 values from oropharyngeal swabs (log10). [file 1317784.f1.zip › supplement tables9.docx]

**Table 9.** Variations between only francolin strains and other genotype Ⅻ NDVs

|  | F | | | | HN | | | NP | L | P | | | | V | | | |
| --- | --- | --- | --- | --- | --- | --- | --- | --- | --- | --- | --- | --- | --- | --- | --- | --- | --- |
|  | 9 | 297 | 411 | 510 | 33 | 36 | 509 | 346 | 122 | 85 | 95 | 101 | 320 | 85 | 95 | 101 | 211 |
| Subtype XIIb (isolates in China) |  |  |  |  |  |  |  |  |  |  |  |  |  |  |  |  |  |
| MZ306226  francolin/China/GX01/2017 | F | R | H | T | I | I | T | S | I | R | S | A | S | R | S | A | P |
| MZ306225  francolin/China/GX02/2017 | F | R | H | T | I | I | T | S | I | R | S | A | S | R | S | A | P |
| MZ306224  Goose/China/GX02/2018 | L | N | N | I | M | T | A | G | T | Q | L | T | G | Q | L | T | S |
| MZ306223  Goose/China/GX17/2018 | L | N | N | I | M | T | A | G | T | Q | L | T | G | Q | L | T | S |
| MK616244  Goose/CH/GD/E115/2017 | L | N | N | I | M | T | A | G | T | H | P | T | G | H | P | T | S |
| JN627504  Goose/GD12/2011 | I | N | N | I | - | - | - | - | - | - | - | - | - | - | - | - | - |
| JN627505  Goose/GD17/2011 | I | N | N | I | - | - | - | - | - | - | - | - | - | - | - | - | - |
| JN627506  Goose/GD20/2011 | I | N | N | I | - | - | - | - | - | - | - | - | - | - | - | - | - |
| JN627507  Goose/GD1003/2010 | I | N | N | I | - | - | - | - | - | - | - | - | - | - | - | - | - |
| JN627508  Goose/GD450/2011 | I | N | N | I | - | - | - | - | - | - | - | - | - | - | - | - | - |
| KC551967  Goose/Guangdong/2010 | I | N | N | I | M | T | A | G | T | H | L | T | G | H | L | T | S |
| Subtype XIIa (isolates in South America)b |  |  |  |  |  |  |  |  |  |  |  |  |  |  |  |  |  |
| KJ865695  Chicken/Colombia/1326-13285/2009 | I | N | N | I | - | - | - | - | - | - | - | - | - | - | - | - | - |
| KJ865696  Chicken/Colombia/1326-13286/2009 | I | N | N | I | - | - | - | - | - | - | - | - | - | - | - | - | - |
| JN800306  Chicken/Peru/1918-03/603/2008 | I | N | N | I | M | T | I | G | T | H | P | T | S | H | P | T | S |
| KR732614  NDV/peacock/Peru/2011 | I | N | N | I | M | T | I | G | T | H | P | T | S | H | P | T | S |
| KU594613  Chicken/Lima-Peru/40931/2004 | I | N | N | I | - | - | - | - | - | - | - | - | - | - | - | - | - |
| KU594614  Chicken/Lima-Peru/40785/2004 | I | N | N | I | - | - | - | - | - | - | - | - | - | - | - | - | - |
| KU594615  Chicken/Apurimac-Peru/50009/2005 | I | N | N | I | - | - | - | - | - | - | - | - | - | - | - | - | - |
| KU594616  Chicken/Lurin-Peru/40871/2004 | I | N | N | I | - | - | - | - | - | - | - | - | - | - | - | - | - |
| KU594617  Chicken/Piura-Peru/60087/2006 | I | N | N | I | - | - | - | - | - | - | - | - | - | - | - | - | - |
| KU594618  Chicken/Arequipa-Peru/VFAR-81/2015 | I | N | N | I | - | - | - | - | - | - | - | - | - | - | - | - | - |
| Subtype XIId (isolates in Vietnam) |  |  |  |  |  |  |  |  |  |  |  |  |  |  |  |  |  |
| MG869270  AAvV1-NDVQG/Chicken/Vietnam/2008 | I | N | N | I | - | - | - | - | - | - | - | - | - | - | - | - | - |
| MG869271  AAvV1-NCXKH/Chicken/Vietnam/2011 | I | N | N | I | - | - | - | - | - | - | - | - | - | - | - | - | - |
| MG869272  AAvV1-NCXMT/Chicken/Vietnam/2014 | I | N | N | I | - | - | - | - | - | - | - | - | - | - | - | - | - |
